# Increasing ventilator surge capacity in COVID 19 pandemic: design, manufacture and in vitro–in vivo testing in anaesthetized healthy pigs of a rapid prototyped mechanical ventilator

**DOI:** 10.1186/s13104-020-05259-z

**Published:** 2020-09-07

**Authors:** Jayesh Dhanani, George Pang, Jason Pincus, Benjamin Ahern, Wendy Goodwin, Nicholas Cowling, Grant Whitten, Mohd. H. Abdul-Aziz, Steven Martin, Peter Corke, Kevin B. Laupland

**Affiliations:** 1 Department of Intensive Care Medicine, Royal Brisbane and Women’s Hospital, Brisbane, QLD Australia; 2 University of Queensland Centre of Clinical Research, Faculty of Medicine, Level 8, The University of Queensland, Herston, QLD 4029 Australia; 3 School of Medicine, University of Queensland, Gatton, Australia; 4 School of Veterinary Science, Faculty of Science, University of Queensland, Gatton, Australia; 5 Australian Centre for Robotic Vision, Queensland University of Technology, Brisbane, Australia; 6 Faculty of Health, Queensland University of Technology, Brisbane, QLD Australia

**Keywords:** Ventilators, Pandemic, COVID 19, Mass disaster, Rapid prototyping, 3 D printing

## Abstract

**Objective:**

The advent of new technologies has made it possible to explore alternative ventilator manufacturing to meet the worldwide shortfall for mechanical ventilators especially in pandemics. We describe a method using rapid prototyping technologies to create an electro-mechanical ventilator in a cost effective, timely manner and provide results of testing using an in vitro–in vivo testing model.

**Results:**

Rapid prototyping technologies (3D printing and 2D cutting) were used to create a modular ventilator. The artificial manual breathing unit (AMBU) bag connected to wall oxygen source using a flow meter was used as air reservoir. Controlled variables include respiratory rate, tidal volume and inspiratory: expiratory (I:E) ratio. In vitro testing and In vivo testing in the pig model demonstrated comparable mechanical efficiency of the test ventilator to that of standard ventilator but showed the material limits of 3D printed gears. Improved gear design resulted in better ventilator durability whilst reducing manufacturing time (< 2-h). The entire cost of manufacture of ventilator was estimated at 300 Australian dollars. A cost-effective novel rapid prototyped ventilator for use in patients with respiratory failure was developed in < 2-h and was effective in anesthetized, healthy pig model.

## Introduction

The current COVID-19 pandemic is responsible for significant mortality worldwide [1].

Due to the combination of being a worldwide event as well as acuity of the disease, there is a limited global capacity to fulfil the increased demand for medical devices [2]. Respiratory failure due to pneumonia and severe respiratory failure requiring mechanical ventilation is one of the features of the disease [3]. However, this has exposed resource limitations to meet the demands for mechanical ventilators [4–7].

Rapid prototyping is a family of manufacturing technologies, including three-dimensional (3D) printing, computer numerical control (CNC) milling and waterjet cutting that generate a physical model from digital information [8]. These technologies offer a viable alternative to rapidly produce medical devices such as ventilators in a high volume and economical manner.

The exact requirements for surge capacity mechanical ventilation are not clear [9]. Some regulatory agencies have described minimum acceptable performance and desired features for the ventilators to be used in select adult COVID-19 patients [10, 11].

In vitro evaluation with test lungs can provide initial data evaluating the mechanical efficacy of the test ventilator [12]. However, large animals like pigs provide in vivo environment to evaluate the safety and efficacy of the test ventilator [13]. The objective of this study was to manufacture and test an economical and rapidly built mechanical ventilator using novel technology for potential use in respiratory failure.

## Main text

### Methods

The study followed an incremental model involving laboratory designing of the ventilator model, detailed bench testing and finally in vivo testing of the ventilator in a large animal model.

#### Ventilator design

Principle- As proposed by Al Husseini et al. [14], the ventilator mimics hand bagging by compression of the bag on one side using a mechanical arm which rotates about a fixed pivot point. The arm is driven by a computer-controlled motor and the machine has just two moving parts.

Design aim was to develop an economical ventilator using as few as possible and easily sourced components in a timely manner. The experimental methods included the manufacturing and testing of a prototype ventilator. Based on preliminary results, changes and retesting of the ventilator were planned if required.

The machine was designed using CAD software (Autodesk Fusion 360). A prototype (version 1) was constructed with CNC cut plywood frame and polylactic acid (PLA) plastic gears.

#### Materials

- The artificial manual breathing unit (AMBU.
- A commercially available stepper motor (Nema 23 with 3Nm of holding torque)
- motor driver (Toshiba TB6560) were powered by a 12 V 5A power supply.
- A microcontroller (Arduino Mega 2560) provided the control software (written in Arduino programming language)
- thin-film transistor (TFT) liquid crystal display (LCD) display (Arduino Shield TFT) for the user interface.

The controllable ventilation parameters are respiratory rate up to 25 per minute; inspiration: expiration ratio (I:E), and tidal volume. A fixed inspiration hold time of 0.1 sec was implemented. Tidal volume, a function of maximum motor rotation, was calibrated by measuring the air expelled from the AMBU bag for single cycles of 20, 40, 80 and 100% of maximum motor rotation. The relationship was found to be quite linear.

#### In vitro study set up

**Fig. 1:**
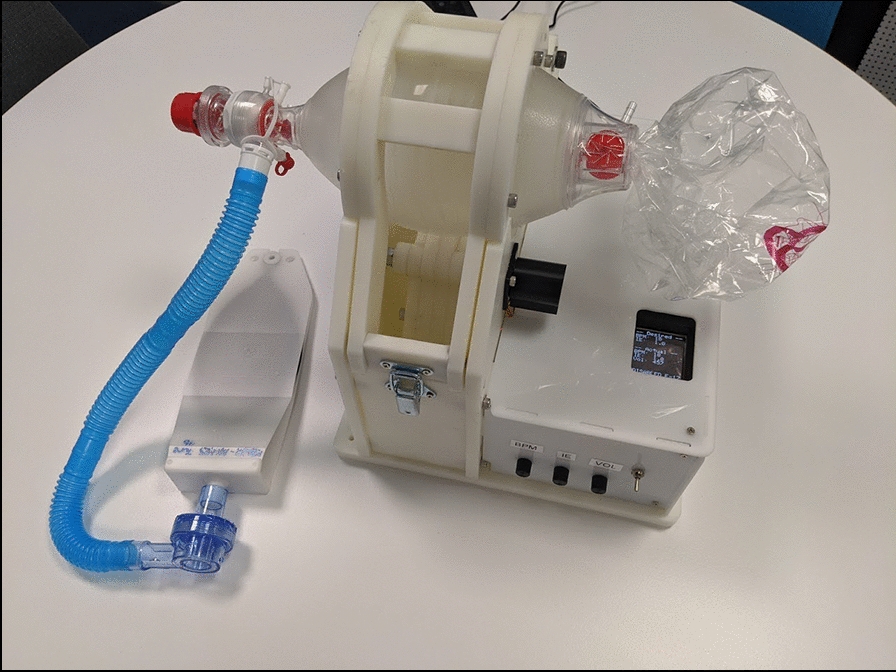
In vitro study set up with test lung and test ventilator

The set-up of the in vitro test is as shown in Fig. 1. The unit included an adult ventilator circuit, the test ventilator and the adult test lung (Dräger Medical, Lubeck, Germany). Using the circuit, the ventilator was connected to the test lung.

##### In vivo study set up

Ethics approval was obtained from The University of Queensland’s Production and Companion Animal Ethics Committee (SVS/142/20).

### Study design

The study was performed using 3 healthy female Large White pigs aged approximately 10 weeks and weighing 35- 40 kg.

### Experimental method

#### Anaesthesia and monitoring

Pigs were anaesthetised and orotracheally intubated. Mechanical ventilation was commenced using a standard ventilator (Ulco Campbell Ventilator EV500, Ulco Engineering Pty Ltd, Marrickville, NSW) with settings (respiratory rate 16 breaths per minute, tidal volume 450 mL, PEEP 5 cm H_2_O, FiO_2_ 0.5). A 22 G (1.4-inch Optiva) catheter was placed in the auricular artery and the urinary bladder catheterised to permit continuous drainage of urine.

The animals were monitored for electrocardiogram, pulse oximetry, invasive blood pressure and esophageal temperature, Fraction of inspired fraction of oxygen (FiO_2_), end tidal carbon dioxide (ETCO_2_) and respiratory rate.

#### Ventilator testing

Each pig was ventilated using the standard ventilator for 12 h and the test ventilator for 12 h (total 24 h). On each ventilator, initial ventilator settings included tidal volume of 500 mL, I:E ratio 1:2 and respiratory rate 16- 18 breaths per minute. PEEP was adjusted on the AMBU bag (test ventilator) or ventilator controls (standard ventilator). PEEP was set at 5, 10 and 15 cm H_2_O each for 4 h whilst on each ventilator. Respiratory rate was adjusted to maintain PaCO_2_ within normal limits and pH > 7.2. Peak airway pressures were maintained < 30 cm H_2_O.

**Fig. 2:**
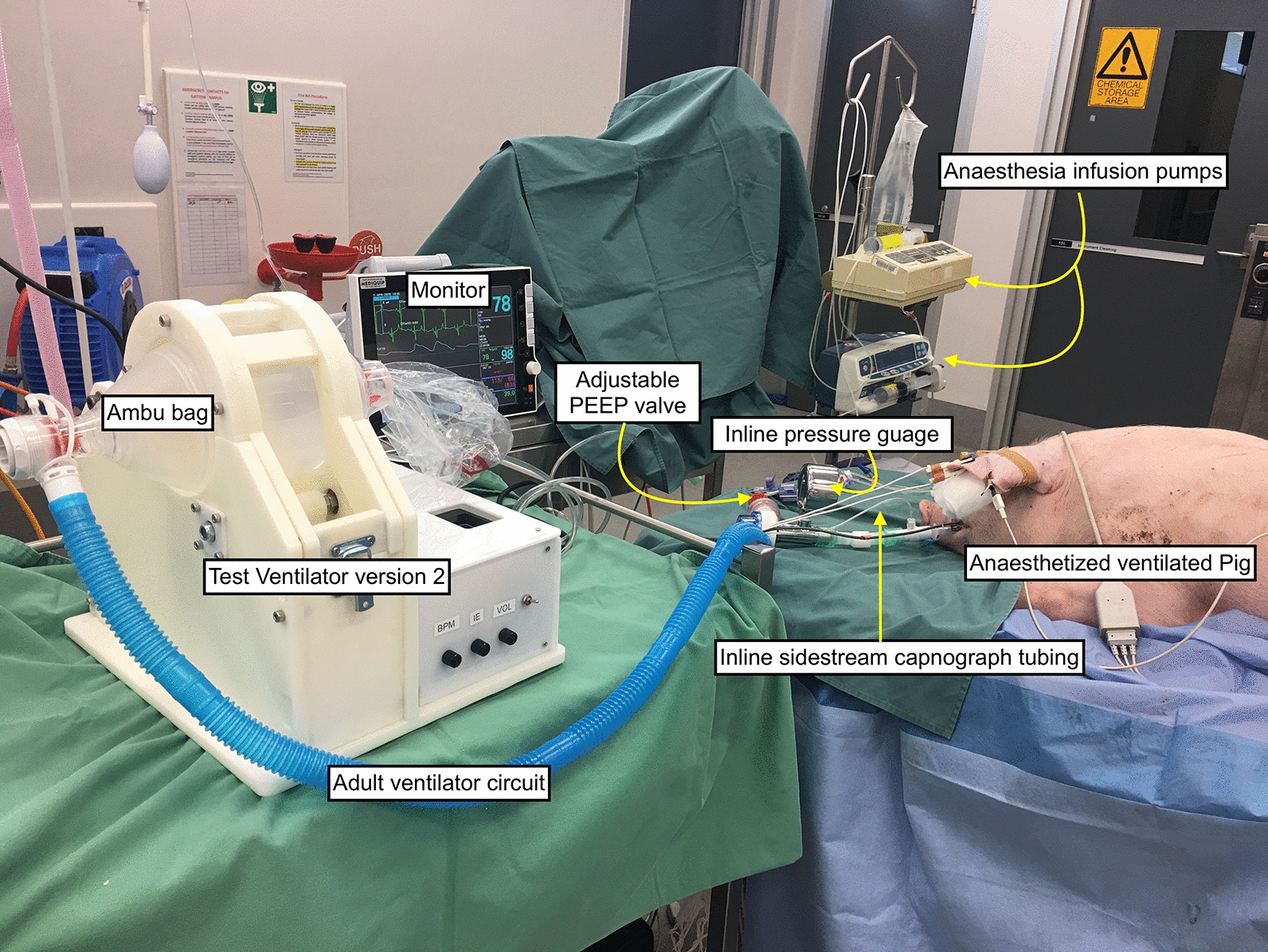
In vivo study method showing operating theater set up, the pig position and instrumentation and monitoring. Pig mechanically ventilated using test ventilator. In line pressure manometer, side stream FiO_2_ and capnography and adjustable PEEP valve

The test ventilator set up is as shown in Fig. 2.

#### Euthanasia

At completion of the experiment, pigs were euthanised using an overdose of intravenous pentobarbitone sodium (Lethabarb Euthanasia Solution®, 325 mg/mL, Virbac Pty Ltd, NSW, Australia).

### Data collection

Arterial blood samples were collected at T = 0 and then at 2 hourly intervals. Arterial samples were immediately analysed using a blood gas analyser (GEM Premier 3500 with iQM®, Instrumentation Laboratory, Lexington MA USA). Vital parameters such as heart rate, blood pressure, ETCO_2_, SpO_2_, and respiratory rate were noted. Ventilator parameters such as set respiratory rate, I:E ratio, tidal volume and peak inspiratory pressure were also recorded. Chest radiographs were obtained at T = 0, T = 12 h and T = 24 h to evaluate the mechanical effects of the ventilators.

### Statistical analysis

Differences of pH, PaO_2_, PaCO_2_, peak inspiratory pressure, tidal volume and respiratory rate at different time-points between standard and test ventilators were analysed using two-way repeated measures ANOVA. A two-sided p-value of < 0.05 was considered statistically significant in all analyses.

### Results

#### Testing and results of version 1

In vitro testing of the prototype (version 1) ventilator was performed using the set up as described earlier. The respiratory rate was 16 breaths per minute, tidal volume 500 mL and I:E ratio at 1:2. PEEP was at 5 cm H_2_O. This set up was successfully tested for endurance and mechanical stability for 12 h.

Following this, an in vivo study was performed using the prototype ventilator in a single pig following the method mentioned above. At high PEEP, the PLA motor pinion softened due to heat from the motor conducted along the motor shaft and disengaged from the D-shaped gear. Urgent replacement with a 3D printed Acrylonitrile Butadiene Styrene motor pinion resulted in a different failure mode where the gear teeth sheared off. Each lasted about 6 h thus completing the 12-h duration on the test ventilator.

#### Development of version 2 ventilator

Following the in vitro and in vivo testing results improvements and changes were made to the prototype ventilator resulting to version 2 of the ventilator. The design aims of version 2 were to redesign the gears by using a heat tolerant material. The gears were made from Nylon (cut from 12 mm sheet using CNC router).

In vitro testing as per version 1 was successfully conducted for 72 h period. The AMBU bag showed some evidence of wear but appeared intact at the end of the test.

Using the study set up used for testing version 1, the in vivo study (Fig. 2) with version 2 was conducted on two pigs (Pig 2 and 3) in a cross over design with each pig simultaneously ventilated using test and the standard ventilator for 12 h (24 h ventilation total).

In vivo study data for the version 1 and version 2 were pooled and analysed. Vital parameters such as temperature, heart rate and blood pressure remained within clinically normal limits throughout the study. Chest X-ray showed no evidence of any complications. The findings were comparable between the standard and test ventilator.

**Fig. 3:**
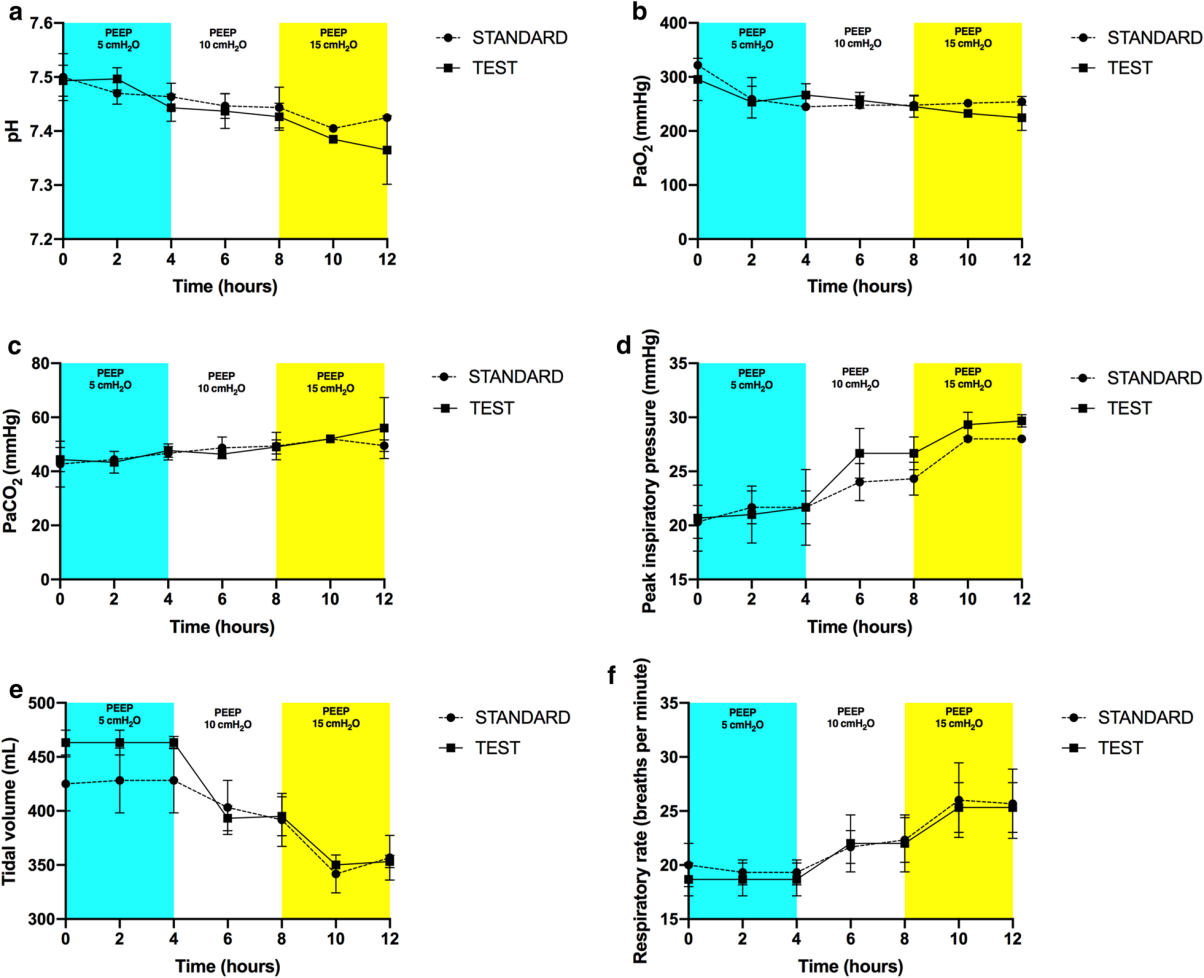
Comparative physiological and ventilator variables between test ventilator and standard ventilator over the 12-h study period for each ventilator for the 3 PEEP settings (5, 10 and 15 cm H_2_O). **a** pH; **b** PaO_2_; **c** PaCO_2_; **d** peak inspiratory pressure; **e** tidal volume; **f** respiratory rate

There were no significant differences in pH, PaO_2_, PaCO_2_, peak inspiratory pressure, tidal volume and respiratory rate at different time-points between standard and test ventilators (p > 0.05) (Fig. 3). To maintain a peak inspiratory pressure ≤ 30 cm H_2_O, at high PEEP (15 cm H_2_O) the tidal volume was reduced and PaCO_2_ increased. Hence respiratory rate was increased to maintain pH > 7.2. However, the peak inspiratory pressure, respiratory rate and tidal volume were comparable between standard and test ventilator. These values remained stable for each PEEP level.

### Discussion

We have successfully demonstrated the design, manufacturing, and testing of a rapid prototyped ventilator that can be used in respiratory failure conditions. The results of the in vivo testing highlight the importance of adjustable respiratory rate and the safety of PEEP titration in a limited function surge ventilator. The study also identified the limitations of 3D printing technology and the materials for use in the manufacture of surge ventilators.

Version 1 of the rapid-prototyped mechanical ventilator showed mechanical efficiency for maintaining stable arterial blood gas parameters without complications.

However, the two material (PLA and ABS) failures demonstrated important limitations that were not obvious during the in vitro tests. This is important as CAD software and 3D printing is a commodity technology and lack knowledge of mechanical design principles (gear design) and material properties may lead to poorly designed mechanical components propagating into designs leading to complications.

The change in the material and design in the Version 2 increased the durability and benefit of rapid fabrication and assembly. It maintained stable arterial blood gas parameters without complications.

PEEP is recommended during the treatment of patients with hypoxic respiratory failure due to COVID 19 pneumonia [15]. Experts have recommended that surge ventilators should be able to provide PEEP [9, 16]. Our model shows that PEEP titration is possible and safe with the test ventilator.

The epidemiology of mechanical ventilation requirements indicates that the initial settings (PEEP, respiratory rate) to be high in patients with COVID 19 and other causes of respiratory failure due to mass disasters [17, 18]. While this level of testing would not be sufficient for use in adult respiratory distress syndrome (ARDS), this type of ventilator would be useful in milder cases or non-COVID 19 patients when there is a shortage of sophisticated ventilators and minimal support is required. Integrating alarms for parameters such as high or low respiratory rate and tidal volume values will provide additional safety.

During surge requirements, short manufacturing time and low cost of a ventilator are important factors, especially for application in low-income countries. 3D printing is also a slow fabrication process for large components whilst the rapid prototyping is a faster process. The short manufacturing time (~ 2 h) and affordable cost (~ $AUD 300) makes the rapid prototyped ventilator a viable alternative.

A growing number of designs for 3D-printed ventilators are appearing but few have been well tested. Clinicians need to be aware of potential weaknesses in these devices. The correct testing standards for surge ventilators should be determined by clinical requirements.

Future studies should consider laboratory-based stress testing of the ventilator (high RR, I:E ratio, tidal volume combined with high PEEP) simulating in vivo conditions prior to animal or human studies.

### Conclusions

In summary, we report the successful development and testing of a low cost, rapid prototyped ventilator. Further improvements in the model and studies in lung injury animal model, for prolonged duration is necessary prior to its application in patients.

## Limitations

Due to the limited function nature of the test ventilator, it provides only controlled mode of ventilation. Although radiological examinations excluded gross lung injury, no histological examinations were performed to definitively exclude microscopic evidence of lung injury. Although in vitro testing for 72 h proved mechanical endurance, the in vivo testing was done for a 12-h period with the test ventilator. Testing for prolonged period may be necessary to establish ventilator efficacy over time. Other parameters of safety such as plateau pressure (alveolar pressure) and intrinsic PEEP were not able to be measured in this ventilator design. This ventilator has not been tested in humans and not in disease conditions such as acute lung injury or acute respiratory distress syndrome.

## Data Availability

The datasets used and/or analysed during the current study are available from the corresponding author on reasonable request.

## Abbreviations

3D: 3 Dimensional
CNC: Computer numerical control
PLA: Polylactic acid
AMBU: Artificial manual breathing unit
TFT: Thin film transistor
LCD: Liquid crystal display
PEEP: Positive end-expiratory pressure
FiO_2_: Fraction of inspired fraction of oxygen
ETCO_2_: End-tidal carbon dioxide
SpO_2_: Oxygen saturation
PaO_2_: Partial pressure of oxygen
PaCO_2_: Partial pressure of carbon dioxide
ABS: Acrylonitrile Butadiene Styrene
ARDS: Adult respiratory distress syndrome
